# Neurobiology of Chronic Pain, Posttraumatic Stress Disorder, and Mild Traumatic Brain Injury

**DOI:** 10.3390/biology14060662

**Published:** 2025-06-07

**Authors:** Gerald Young, Hella Thielen, Kristin Samuelson, Joel Jin

**Affiliations:** 1 Department of Psychology, Glendon College, York University, 2275 Bayview Avenue, Toronto, ON M4N 3M6, Canada; 2 Department Brain & Cognition, Leuven Brain Institute, KU Leuven, Tiensestraat 102—Box 3711, 3000 Leuven, Belgium; hella.thielen@kuleuven.be; 3 Private Practice, New Orleans, LA 70112, USA; kristinsamuelsonphd@gmail.com; 4 Department of Family Medicine, University of Washington, 314 NE Thornton Place, Box 356390, Seattle, WA 98125, USA; joeljin@uw.edu

**Keywords:** neurobiology, sensitization, chronic pain, mild traumatic brain injury, posttraumatic stress disorder

## Abstract

Objectives: This article describes the neurobiology of psychological injuries—chronic pain, concussion/mild traumatic brain injury (MTBI), and fear/posttraumatic stress disorder (PTSD)—toward elucidating common mechanisms in central and peripheral sensitization that contribute to their onset, exacerbation, and maintenance. Central sensitization refers to central nervous system (CNS) and related processes, while peripheral sensitization is typically referred to as receptor field expansion. The three psychological injury diagnoses/conditions are accompanied by impairments in function after negligent events (such as motor vehicle accidents (MVAs)) that lead to tort court action. Methods: The conducted literature review involved an extensive scoping review of recent neurobiological literature on chronic pain, PTSD, and MTBI. The literature review sought biological markers that distinguish them. Results: For chronic pain, concussion/MTBI, and fear/PTSD, this article reviewed definitions and critical neurobiological research. The literature review did not find evidence of biological markers, but the role of sensitization emerged as important. Conclusions: Common therapeutic processes, such as focusing on sensitization, might be helpful for these conditions. As for causal mechanisms related to sensitization in the causality of psychological injuries, the major ones hypothesized relate to the biopsychosocial model, psychological control, and activation–inhibition coordination.

## Simple Summary

This article describes the neurobiology of mild traumatic brain injury, posttraumatic stress disorder, and chronic pain. It develops a common framework linking them in central and peripheral sensitization. It reviews the literature for possible biomarkers of these conditions, but none are found. This article seeks common explanatory mechanisms in other models, such as the biopsychosocial model, the concepts of psychological reserve and psychological control, and the concept of activation–inhibition coordination. These factors work at different levels or scales of the brain–behavior interface. This article helps researchers and practitioners appreciate the complexity of the conditions of mild traumatic brain injury, posttraumatic stress disorder, and chronic pain.

## 1. Background Works

This article describes the neurobiological bases in the expression of psychological injuries (especially chronic pain, concussion/mild traumatic brain injury (MTBI), and fear reactions/posttraumatic stress disorder (PTSD)) after events at claim (e.g., accidents leading to tort). The literature review on the topic found common descriptions for the psychological injuries related to sensitization, both at the peripheral and central levels. Sensitization might be part of the common mechanisms that are involved in the establishment, as well as the possible maintenance and worsening, of the symptoms of psychological injuries. This article reviews the psychological injury conditions, presents recent literature on their neurobiology, and discusses practical applications, including in terms of sensitization. This article concludes with a causal model integrating sensitization in the psychological injuries.

The literature review focused on recent articles in a broad survey of neurobiology, seeking biomarkers for the three diagnoses/conditions at issue. Multiple data engine searches were used throughout 2023–2024 with multiple terms related to the multiple topics of this article that are too numerous to list here. We did not use artificial intelligence in the search, given the lack of access to AI to scientific articles behind pay walls, which would lead to incomplete lists of critical articles, including on any biomarkers. Biomarkers help specify the presence of psychological injuries in injured patients, and their presence could be critical in proper diagnosis and assessment. In this sense, the literature was not comprehensive of every critical study in the area over decades but focused on recent ones in particular. Nor did the review attempt to canvas all possible cellular, neuronal, and brain circuitry findings for the psychological injuries, instead only determining which ones were highlighted in the recent literature.

This article is structured into five major sections, with the first three on the neurobiology of psychological injuries—chronic pain, MTBI/concussion, and fear/PTSD. Each of the three injuries are first defined, and then critical research related to their neurobiology, including with respect to sensitization, is offered. Then, this article delves deeper into causation and sensitization, ending with discussion and implications. This article uses summary tables to simplify the dense conceptualization and research findings.

Chronic pain is a condition, not a psychological diagnosis, but it is an example of the Diagnostic and Statistical Manual of Mental Disorders, 5th ed., Text Revision (DSM-5-TR) diagnosis of somatic symptom disorder (SSD) with predominant pain [1]. Neither MTBI nor concussion are psychological diagnoses in that they are conditions of interest. The more important condition in the field of psychological injury and law concerns PPCS, which is reviewed herein as well. In cases of trauma, there are several trauma-related diagnoses that can apply, but PTSD is the most pervasive one. In addition, the literature review considers the topic of fear in relation to trauma when it applies. There are other conditions and diagnoses, including depression, that could result after negligent events, such as motor vehicle accidents (MVAs), but we focus on the three major ones that are considered contentious in the field [2].

The concept of central and peripheral sensitization originated with work on pain experience and conditions. Indeed, the original of these concepts lie in the chronic pain literature [3]. The definition of chronic pain is consistent with the influence of both central and peripheral factors in the symptomology of pain and its maintenance/exacerbation into the chronic state. The generalization in the model to fear/PTSD and MTBI/concussion of the sensitization mechanism is consistent with the literature review, which includes aspects of sensitization in all three conditions.

## 2. Neurobiology

### 2.1. Chronic Pain Sensitization

#### 2.1.1. Definitions

The definition of pain has been updated by the International Association for the Study of Pain (IASP) [4]. The recent IASP definition emphasizes the biopsychosocial context of the expression of pain and includes reference to tissue injury. Well-described apparent musculoskeletal conditions for which the causes are incompletely understood, such as nonspecific back pain or chronic widespread pain, are included in the IASP section on “chronic primary pain” [5].

Fitzcharles et al. described three types of pain conditions [6]—the more traditional nociceptive and neuropathic pain and the recently introduced nociplastic pain. Nociceptive pain is related to inflammation and an ongoing input from tissue damage, either real or threatened, while neuropathic pain is related to nerve damage, especially in the case of injury or disease impacting the central nervous system (CNS) or peripheral nervous system (PNS). Nociceptive symptoms are more widespread compared with the other types (multifocal). Its symptoms normally are greater than would be expected given the assessed amount of identifiable nerve or tissue damage. In addition, there could be associated symptoms that do not accompany other pain categories, such as fatigue, sleep, and mood problems. Indeed, one recommendation has been to create the nomenclature of “nociplastic pain syndrome”. The mechanisms postulated for nociplastic pain include increased or altered CNS activity and pain-related sensory processing/pathways, as well as altered pain modulation [3], with cumulative biopsychosocial factors involved as well. The descriptor is used in cases of chronic pain in which there is indication of altered nociceptive function but no obvious activation of nociceptors or signs of neuropathy [7]. The pain mechanisms can be considered top–down (central) and bottom–up (peripheral) in dynamic interaction, resulting in one or both of (a) augmented signal processing or (b) a reduced inhibition of pain related stimuli at multiple levels in the nervous system. As specifically as indicated by Kosek et al. [7], the pain in nociplastic pain “arises from altered nociception” even though there is no “actual or threatened tissue damage” that is responsible for “causing the activation peripheral nociceptors”, aside from no evidence for the presence of “disease or lesion in the somatosensory system” (Sterling and Davis, 2024; [8] (p. S1). Buldyś et al. (2024) concurred that nociplastic pain includes psychological aspects [9].

Fitzcharles et al. described that the IASP categorization of pain included subdivisions of chronic primary pain [6]. The latter is defined as chronic pain experienced in at least one anatomic region and it is associated with “notable” emotional distress/disability. In addition, the experienced pain cannot be explained by other conditions. Chronic primary pain subcategories include primary musculoskeletal pain, chronic widespread pain (e.g., fibromyalgia), complex regional pain syndrome (CRPS), and ones related to headaches, orofacial pain, and visceral pain.

Fitzcharles et al. graphed the mechanisms and features of nociplastic pain [6]. Features include combined central/peripheral causation, hyperresponsivity to signals even if none are present, and anxiety and depression. Supraspinal mechanisms include hyperactivity/connectivity among pain-related brain regions, decreased descending pathway inhibitory activity, and grey and white matter changes. Spinal mechanisms include “regional clustering and convergence of signals from different from different pain loci” [6] (p. 2100), amplified transition in spinal reflexes, and reduced spinal inhibition. Peripheral features include elevated cytokine and chemokine concentrations among changes related to peripheral sensitization, which include the expansion of peripheral receptive fields. In practice, the three types of pain (nociceptive, neuropathic, and nociplastic) often are mixed, as might occur in cases of chronic low back pain.

#### 2.1.2. Critical Research

##### Sensitization

Volcheck et al. reviewed the role of central sensitization in chronic pain [10]. These authors referred to it as a pathological process in which the processing of pain is altered, rendering the patient more sensitive to pain and other sensory input while acting to increase fatigue and related symptoms as well. There are structural, chemical, and functional changes in the CNS that take place and lead to heightened neural reactivity.

The range of structural, chemical, and functional changes that mark central sensitization is extensive, and Volcheck et al. listed these changes in their Table 1 [10]. Among these factors in central sensitization-based chronic pain, the following ones are notable: (a) The presence of cell membrane hyperexcitability in CNS neurons, along with a decreased action potential threshold. The synapses increase in strength. There is decreased descending inhibitory transmission. The nerves involved have a reduced activation threshold and “enlarged receptive fields” [10] (p. 246). [This shows that central sensitization has peripheral sensitizing effects.] (b) There is an increased temporal summation of nociceptive signals. This is accompanied by an increased ascending sensory amplification of the signals and a concomitant reduction in descending inhibitory signals from higher levels in the involved pathway. (c) The neurotransmitters in the process are more concentrated in the cerebrospinal fluid. (d) There is maladaptive peripheral and central neuroplasticity (neuropathy). (e) There are associated hypothalamic–pituitary–adrenal (HPA) axis changes. (f) There are central structural and functional changes in the thalamus, hypothalamus, and amygdala. (g) Functional activity is accentuated in the somatosensory cortex and insula. (h) The cingulate cortex and prefrontal cortex lose grey matter. (i) The sympathetic nervous system, part of the autonomic nervous system (SNS and ANS, respectively) becomes hyperactive, as does the endogenous opioid system.

Volcheck et al. explained that the gamut of changes in central sensitization could even lead to pain experience without sensory input [10]: the pain experienced can be widespread (disparate) or beyond (migratory, incongruent) the original location of the experienced pain. There is a trifecta in the pain experience: with hyperalgesia (the painful stimulus becomes associated with even more pain [10] (p. 246), allodydynia (previously nonpainful stimuli come to evoke pain experience, e.g., a light touch), and global sensory hyperresponsiveness (e.g., to bright lights). Because the pain experience can be influenced by psychological factors in chronic pain patients, Volcheck et al. touted the role of psychotherapy, such as in cognitive behavior therapy in dealing with chronic pain (e.g., turning negative and distorted beliefs into positive and rational ones) [10]. Other interventions include Mindfulness-Based Interventions (MBIs) [11], Acceptance and Commitment Therapy (ACT) [12,13], and Emotional Awareness and Expression Therapy (EAET) [14].

Martin et al. conducted a study of primary total knee arthroplasty (TKA) patients both before and after surgery (6 months later) using psychological, pain, and central sensitization measures [15]. The latter included a measure of the temporal summation of (knee) pain (TSP) using repeated Frey stimuli sensitivity testing. They conducted a cluster analysis that found three types of patients characterized by the following: (a) low psychological distress, low hyperalgesia, and low TSP; (b) elevated psychological distress, low hyperalgesia, and high TSP (high risk for central sensitization); and (c) elevated psychological distress and hyperalgesia but without elevated TSP (high risk for peripheral sensitization). As for the 6-month outcome, the central group was the only one with worse surgery outcomes while also having more widespread pain.

##### Other Neurobiology

A review of the neurobiology of chronic pain gives further insight into the sensitization processes involved in its development, maintenance and propagation. Baller and Ross diagrammed the pathway in the development of chronic pain from the initial receptor experience to its embedding in the brain [16]. Baller and Ross depicted the pain signaling pathway as involving afferent and efferent ones, with the descending ones capable of inhibitory action on lower levels of the system (or exacerbatory actions) [16]. The afferent pathways include sensory fiber synapses in the dorsal horn of the spinal cord, the spinothalamic tract, and then the cortex. Chronic pain causes the remodeling and rewiring of the nervous system. It could lead to aberrant sprouting and connections in the dorsal horn, facilitating pain experience that normally would not derive from certain sensory inputs. The synapses become sensitized in the neuronal firing, and some of the signaling mechanisms released in prolonged C fiber firing include alterations in presynaptic glutamate, postsynaptic AMPA receptors, depolarization of postsynaptic neurons, and alterations of related magnesium and calcium flow and flux in axons.

Li et al. reviewed the literature on brain alterations associated with chronic low back pain [17]. They documented changes in white matter, grey matter, and neural circuits, as well as structural and functional connectivity abnormalities. For grey matter, some of the affected areas include the nucleus accumbens (NA), the anterior cingulate cortex (ACC), and the secondary somatosensory cortex. For white matter, among others, the authors referred to the medial and lateral prefrontal cortices. Kinsces et al. referred to the brain regions of the amygdala, posterior insula, sensorimotor region, frontoparietal region, and cerebellum in pain-related learning; Ferraro et al. referred to dysregulated insula function [18,19]. At a network level, the resting state cingulo–frontal–parietal network has been implicated in chronic back pain [20]. Mohapatra et al. referred to genomic signatures in chronic pain patients, mostly related to inflammation [21].

#### 2.1.3. Interim Conclusion

Table 1 summarizes the literature review’s major findings on the neurobiology of chronic pain. Aside from the peripheral sensitization findings, it shows that the central regions and circuitry, from the spinal cord to the frontal cortex and associated connections, are widespread in chronic pain.

For neuropathic pain and for complex regional pain syndrome, the contributions of psychological aspects were discussed by Viera et al. and Devarajan et al., respectively [22,23]. Similarly, in pain–sensorimotor interactions, Murray and Sessle referred to the biopsychosocial model [24]. The approach of the DSM-5-TR to diagnosing pain specifies the use of the term somatic symptom disorder (SSD) with predominant pain as a focus [1]. Fitzcharles et al. indicated that this disorder is psychological at baseline and independent of any disease process involved in contributing to the pain experience [6].

Forensic psychologists who conduct disability assessments know that chronic pain conditions have a psychological component. This section of the paper provides details on the neurobiology of chronic pain, including for encountered conditions, such as low back pain, to give focus to the biological and neurological underpinnings involved. The evidence indicates that chronic pain experience is a physiological and neurological process, aside from evident psychological involvement, in a complex whole-person perspective. From a forensic viewpoint, the possible creation of chronic pain experience without known tissue damage is controversial and would not be accepted in court as a valid reason for chronic pain after a negligent event purportedly leading to it.

As with the ensuing sections on MTBI and PTSD, the absence of confirmed biomarkers for chronic pain works against qualifying survivors of negligent events as having an uncontestable psychological injury for individual cases at hand. Therefore, the forensic disability assessor must conduct comprehensive, scientifically informed, and unbiased assessments, including the use of validity/credibility tests for court purposes [2]. That said, research needs to be conducted on the neurobiology of bona fide chronic pain patients compared with those with those with invalid response set and feigning/malingering included in research samples.

### 2.2. Concussion/MTBI Sensitization

#### 2.2.1. Definitions

The definition and diagnostic symptoms of MTBI have recently been upgraded by the American Congress of Rehabilitation Medicine (ACRM) [25]. MTBI is defined as including (i) a loss of consciousness (LOC) duration of less than 30 min; (ii) after 30 min, a Glasgow Coma Scale (GCS) of 13–15; (iii) post-traumatic amnesia (PTA) of less than 24 h; and (iv) a Neuroimaging Qualifier: if abnormal, MTBI can be qualified as including “neuroimaging evidence of structural intracranial injury”. The term concussion can be used for MTBI when neuroimaging is normal or not clinically indicated.

Silverberg et al. divided their list of MTBI symptoms into the categories of cognition, physical symptoms, and emotional symptoms [25]. The list in their Box 2 of the symptoms included the following: (a) cognition—confused, disoriented, dazed, feeling slowed down, mental fog, difficulty concentrating, memory problems; (b) physical—headache, nausea, dizziness, balance problems, vision problems, light sensitivity, noise sensitivity; (c) emotional—irritability, atypical emotional lability. The listing excludes criteria, such as length of retrograde amnesia and loss of consciousness, found in the official ACRM criteria of MTBI.

Heightened sensory sensitivity is common after MTBI, but research is scant due to a lack of proper measures and methodology [26,27]. To address this gap, Thielen et al. developed the Multi-Modal Evaluation of Sensory Sensitivity (MESSY), a sensory sensitivity questionnaire designed for use after MTBI and other degrees of acquired brain injury [26]. The questionnaire examines sensory sensitivity across multiple domains (auditory, visual, tactile, olfactory/gustatory (chemosensory sensitivity), motion sensitivity, and environmental temperature sensitivity), and it is standardized. Using the MESSY, Thielen et al. discovered that post-MTBI sensory hypersensitivity is present across different modalities and that symptom severity does not significantly differ based on TBI severity [26].

#### 2.2.2. Critical Research

##### Sensitization

Despite its clinical relevance, research into the underlying mechanisms of post-MTBI sensory hypersensitivity remains limited. A possible underlying mechanism is sensory sensitization, which involves a neural or behavioral over-responsiveness to sensory stimuli. It is important to distinguish between sensory sensitization and sensory hypersensitivity. Sensory hypersensitivity refers to the subjective experience of heightened sensitivity to sensory stimuli after MTBI as compared with pre-injury levels. This post-injury increase in sensory sensitivity is potentially linked to sensory sensitization. The relationship between sensory sensitivity and sensory sensitization can be conceptualized across different levels of analysis: neuron, brain and behavior.

On a cellular level, MTBI might disrupt the balance between the inhibitory neurotransmitter gamma-aminobutyric acid (GABA) and the excitatory neurotransmitter glutamate. Studies in rodent models suggest that this imbalance is driven by a post-injury release of excitatory neurotransmitters including glutamate, a buildup of glutamate in the synapses due to the disruption of the glutamate transporters, damage to GABA-producing cells, and alterations in glutamate and GABA receptors [28,29]. This GABA-glutamate imbalance increases brain excitability, which may contribute to heightened arousal and attentiveness to the sensory environment, which in turn might lead to complaints of sensory hypersensitivity. Thomas et al. demonstrated that in rodents, higher glutamate release after MTBI was related to sensory hypersensitivity symptoms [30]. However, direct evidence linking glutamate–GABA imbalance to sensory sensitivities in humans is lacking.

In addition to cellular processes, cortical hyperexcitability and functional abnormalities are potentially implicated in sensory hypersensitivity. A study by Astafiev et al. found increased neural responsiveness in sensory areas to sensory stimulation in MTBI patients with light hypersensitivity [31]. Beyond regional abnormalities, functional disruptions in large-scale neural networks, such as the salience network and the default mode network (DMN), may also play a role. The salience network, which comprises the insula, amygdala and anterior cingulate cortex, is responsible for directing attentional resources towards relevant and salient stimuli, as well as assigning emotional valence to both internal and external stimuli [32]. Abnormalities in this network may result in misdirected attention to irrelevant stimuli, overwhelming limited attentional resources and thereby contributing to sensory hypersensitivity. Additionally, salience network abnormalities might lead to improperly assigning negative emotional valence to neutral sensory stimuli, which can again exacerbate hypersensitivity symptoms. The salience network also plays a key role in modulating the activity of other large neural networks, including the DMN. The DMN, which comprises the posterior cingulate cortex, precuneus, medial prefrontal cortex and angular gyrus, is primarily involved in internally oriented, self-referential processing during rest (Buckner et al., 2008) [33,34,35]. Dysfunctional DMN activity, particularly its failure to deactivate, can heighten focus on external stimuli and reduce inhibitory control, which may contribute to sensory hypersensitivity [36]. Evidence indicates heightened salience network–DMN coupling and connectivity abnormalities within the salience network and DMN in individuals with MTBI [37,38,39,40]. However, the direct link between these abnormalities and post-MTBI sensory hypersensitivity has yet to be established.

##### Other Neurobiology

The pathophysiology of MTBI especially involves diffuse axonal injury (DAI) and an associated metabolic cascade. These effects are temporary and resolve, for example, within hours to days, so they cannot account for persisting symptoms. According to Jang, DAI refers to the microscopic axonal damage produced by mechanical forces (the sudden acceleration, deceleration, or rotation of the brain), resulting in axonal stretching, disruption, and the eventual separation of nerve fibers, mainly in areas at the border between the cortical gray and white matters, such as in the corpus callosum, brainstem, and cerebellum [41].

Current neurobiological research on MTBI/concussion is scant, but some findings illustrate possible biomarkers (Beard et al., 2024), although with nothing definitive (Bigler, 2023) [42,43]. Beard et al. (2024) reviewed the literature with respect to the following types of possible biomarkers of MTBI: fluid biomarkers (astrocyte-derived, neuron-derived, neurofilaments, amyloid-related), inflammatory biomarkers, extra-cellular vesicles, imaging biomarkers (structural and functional), and physiological biomarkers (balance/vestibular, visual dysfunction, autonomic) [42]. Centrally, Irimia et al. (2022), Papini et al. (2024), and Wade et al. (2024) referred to altered white matter [44,45,46]. Clarke et al. (2024) referred to inflammation markers [47]. Klimova et al. (2022) referred to altered functional connectome in MTBI, with similar results for PTSD [48]. The areas involved included reduced connectivity in the DMN, the executive control network, and the visual–auditory networks. Additionally, the study found hyperconnectivity across the dorsal/ventral attention networks and perceptual regions. For pediatric and elderly populations, Onicas et al. (2024) and Mahoney et al. (2022) referred to the functional connectome and cortical demyelination, respectively [49,50]. Other connectome and neuropsychological research has been conducted with military veterans (Rowland et al., 2024; de Sousa et al., 2024; respectively) [51,52].

#### 2.2.3. Interim Conclusion

Table 2 summarizes the literature review’s major findings on the neurobiology of MTBI/concussion. Beyond the sensitization findings on sensory sensitization, it shows that the integrated intracortical neurocircuitry are especially involved in MTBI/concussion, along with perceptual regions.

On a behavioral level, Shepherd et al. proposed that subjective sensory hypersensitivity might be due to an overarousal of the SNS related to sensory hypervigilance and threat-monitoring [53]. These mechanisms parallel ones proposed in PTSD. Supporting evidence for this hypothesis is found in studies demonstrating a strong relationship between sensory hypersensitivity and anxiety and post-traumatic stress in people with MTBI [53,54,55]. Additionally, elevated heart rates observed in MTBI patients with sensory hypersensitivity provide further support [56].

Another psychological framework, the fear-avoidance model, originally developed to explain persistent chronic pain—has been applied to persistent MTBI symptomatology [57,58,59,60]. Applied to sensory hypersensitivity after MTBI, this model posits that interpreting sensory stimuli as harmful or threatening could trigger sensory-related fear and avoidance behaviors. Avoidance, in turn, prevents the sensory system from habituating to sensory stimuli, exacerbating sensory sensitization and perpetuating symptoms over time. However, these models have yet to be empirically tested in MTBI patients with sensory hypersensitivity.

At the forensic level, in complicated MTBI, there is evidence of cognitive effects, although the reviewed studies did not screen for invalid response set/feigning or malingering. Similar validity concerns are raised for research on vestibular effects (Aljabari et al., 2024) [61].

### 2.3. Fear/PTSD Sensitization

#### 2.3.1. Definitions

Per the DSM-5-TR, there are four clusters of PTSD symptoms [1]. The entry criterion is having had experienced a traumatic event, aside from functionality/impairments related to the effects of the posttraumatic symptoms. The four symptom clusters involve (a) re-experiencing/intrusions, (b) avoidance, (c) alterations in thought and mood, and (d) arousal/threat. There are two other dissociative symptoms that might apply for a dissociative subtype. To receive the diagnosis, the person needs to manifest at least one symptom in each of clusters a and b and at least two symptoms in each of clusters c and d. The DSM-5-TR symptom list is polythetic, meaning that not all symptoms in each of the four clusters need to be experienced to receive the PTSD diagnosis.

Next, the article authors examine the more restrictive symptom list of PTSD in the ICD-11 [62]. Also, we describe the added criteria for Complex Posttraumatic Stress Disorder (CPTSD) in the ICD-11 [63]. In the ICD-11, PTSD symptoms are clustered into three categories: re-experiencing in the present the traumatic event at issue, avoiding traumatic reminders tied to the traumatic event, and experiencing persistent perceptions of a heightened current threat. CPTSD adds three additional symptoms related to pervasive, cross-context disturbances in self-organization (DSO): difficulties in emotional regulation (e.g., dysregulation in calming down, irritability), having a negative self-concept/negative beliefs about oneself (e.g., feeling worthless), and having relationship/interpersonal problems (e.g., avoiding relationships, feeling distant from others).

Although not a requirement for diagnosis, CPTSD often emerges following long-term traumas, such as from early life adversities and [64,65]. The International Trauma Questionnaire (ITQ) is a well-validated measure of CPTSD symptoms [66].

#### 2.3.2. Critical Research

##### Sensitization

The article by Rosen and Schulkin reviewed next indicates how fear triggers are liable to perceptual sensitization [67]. In addition, it describes important ways that underlying psychobiological activation and inhibition function in fear, anxiety, stress, and trauma symptom expression. The article applies well to the case of PTSD.

Rosen and Schulkin described hyperexcitability in fear that leads to anxiety and trauma reactions [67]. They focused on how normal fear reactions can become pathological. They coined the term “perceptual fear response” (the perception of danger and subsequent fear response) [67] (p. 1) and described how it is overactivated and becomes super hyperresponsive in PTSD. There is hyperactivity in both disinhibitory and inhibitory processes interacting with excitatory neurons that is induced by sensitization. The hyperexcitability is found in the amygdala, and the inhibitory effects are found in the hippocampi and prefrontal cortex. Somatostatin is the neuropeptide involved in the latter, with the excitatory neuropeptide being corticotrophin releasing factor. The prefrontal, insular, and cingulate cortices interact with the amygdala in PTSD. Chronic and acute stress create hyperexcitability in neurons in the lateral nucleus of the amygdala. There are also reduced action potential thresholds due to the “loss of calcium-activated potassium channel hyperpolarization” [67] (p. 5). Somatostatin GABAergic interneurons are part of interneurons that create a broad inhibitory blanket over excitatory principal cells located in the cortex, amygdala, and hippocampus, per animal research [67] (p. 7). In exaggerated fear, the research suggests that the “loss of somatostatinergic inhibitory interneurons” acts to disinhibit principal neurons, which leads to increases in their “excitability and sensitivity” [67] (p. 8). Consequently, mild threats have a disproportionate effect, leading to enhanced perception and fear responses.

Fleming et al. referred to sensory alterations in PTSD [68]. Sensory modulation processes in PTSD act to increase sensorimotor cortex activity in response to stimuli in modality-specific ways (e.g., in hypo-arousal, hyperresponsivity to triggers, and physiological flashback concomitants). Altered white matter and connectivity findings in PTSD, including in dynamic functional connectivity related to the visual network (also see Chou et al., 2024, and Harnett et al., 2024), support the sensory bias in perceptions in PTSD [69,70].

Maria-Rios and Morrow referred to “cue reactivity” in PTSD in these regards [71]. Cue reactivity refers to cross-scale enhanced neuronal, emotional, and motivational response to stimuli associated with salient events. Maria-Ross and Morrow related PTSD symptomatology to dopaminergic activity, for example, in the ventral tegmental area, the midbrain, the striatum, and the prefrontal cortex [71]. PTSD patients have been shown to have an increased density of striatum-located dopamine transporters. Acute stress exposure leads to the release of dopamine in the NA, which appears to be mediated by the activation of the HPA axis.

##### Other Neurobiology

Further current neurobiological research adds to understanding of the physiological/biological/neurological bases for PTSD. Shalev et al. reviewed the neurobiology of PTSD [72]. Genetic risk factors included polymorphisms related to FKBP5, PACAPI1, COMT, DRD2, GABA, and RSG2, among others. The involved fear conditioning system includes multiple components in the stress response, including in the HPA axis. Central regions include the amygdala, hippocampus, insula, and prefrontal structures. Threat detection and emotion regulation circuits include the dorsal anterior cingulate and insular cortices, hippocampus, and prefrontal cortex (PFC) regions.

In PTSD, the DMN and the central executive network (CEN) have been found to be underactive while the salience network is overactive. For research on altered functional connectome activity in PTSD, refer to Beukelaar et al. (2021), Dai et al. (2023), and Suo et al. (2023) [73,74,75]. For work on particular brain regions in PTSD, refer to Davis and Hammer (2024, amygdala), Haris et al. (2023, amygdala), Huggins et al. (2024, cerebellum), Korem et al. (2024, limbic system), McCall et al. (2024, locus coeruleus), Szeskzo et al. (2022, hippocampus), Venkataraman and Dias (2023, thalamus), and Zhong et al. (2024, cerebellum) [76,77,78,79,80,81,82,83]. For heart–brain axis research, refer to Li et al. (2024) and Seligoski et al. (2024) [16,84]. For stress-related work, refer to Cardoner et al. (2024) [85]. For inflammation research, refer to Dmytriv et al. (2023) and Patas et al. (2024) [86,87]. For genetic research, see Dahrendorff et al. (2023) and Nieurgelt et al. (2024) [88,89]. Other relevant research concerns comorbidity (Bommaraju et al., 2024), sex differences (Borst et al., 2024), and self (psychological) issues (Agathos et al., 2024; Lidell et al., 2024) [90,91,92,93].

#### 2.3.3. Interim Conclusion

Table 3 summarizes the literature review’s major findings on the neurobiology of fear/PTSD. Other than the sensitization findings, which implicate perceptual sensitization, it shows that PTSD has biological roots in widespread central activity, from relay stations to the limbic system, cerebellum, frontal areas and network circuitry.

These network irregularities underlie psychiatric symptoms and cognitive difficulties observed in PTSD. In terms of PTSD symptomatology, the overactive salience network contributes to hypervigilance and threat sensitivity while the underactive DMN and CEN contribute to failure to inhibit those responses, emotion dysregulation, and working memory deficits. Inhibitory control deficits are often considered the most clinically significant cognitive impairment related to PTSD because failure to gate information and inhibit impulses influences how individuals process, encode, and retrieve trauma memories (Aupperle et al., 2012) [94]. This process contributes to heightened reexperiencing symptoms. In PTSD, inhibitory control appears to worsen when trauma-relevant information is present (Bomyea et al., 2017; Cisler et al., 2011) and when there is a disproportionate degree of attentional resources directed towards threat-related stimuli or cues (Constans, 2005; Fani et al., 2012) [95,96,97,98]. However, these attention biases towards perceived threats are maladaptive in the absence of real threats and serve to maintain PTSD symptoms by disrupting the ability to learn from safety cues in the environment (Bomyea et al., 2017; Liberzon and Abelson, 2016) while also reinforcing negative cognitions (Elhers and Clark, 2000; Fani et al., 2012) [95,98,99,100].

The research on the neurobiological underpinnings to PTSD is robust and ongoing. It should dispel the notion in the forensic context that it is a uniquely psychological condition.

## 3. Summary

The literature review on the neurobiology of chronic pain, MTBI, and PTSD has shown the multiple biological influences and factors at play in psychological injuries. These factors include two interacting sensitization processes that are associated with chronic pain but are also evident in MTBI and PTSD to some degree (central and peripheral sensitization) [10]. Respectively, central and peripheral sensitization refer to (a) neurons/neuronal networks that are more easily activated and expand in the brain and (b) bodily processes with similar properties (e.g., receptor sensitization nociceptors in pain experience). Chronic pain is traditionally associated with receptor field sensitization. Here, it is posited that fear/PTSD is more associated with perceptual sensitization while the effects of MTBI/concussion are more associated with sensory sensitization. To generalize from pain-related nociception and peripheral sensitization, this would refer to somatization in general in various persistent post-concussion syndrome (PPCS) symptoms (e.g., headaches, dizziness). Also, it would refer to the perceptual priming/hypervigilant response in PTSD.

The next part of this article presents a causal model on the factors involved in PTSD, chronic pain, and concussion/MTBI. The model is a generic one for all behavior but is applied to the major psychological injuries. First, we examine broad models, such as the biopsychosocial model and models involving scales across the central and behavioral systems. Then, we elaborate on a sequence of input processing in terms of: (a) interlinked, reciprocally dynamic reception, sensation, perception, and conception; (b) the nature of psychological control in the integrated causal system, and; (c) the fundamental role of activation–inhibition coordination across the multiple scales of the system. The concepts of peripheral and central sensitization in chronic pain, MTBI/concussion, and fear/PTSD fit squarely in the biological component of the biopsychosocial model but with exacerbations possible from psychological and social factors.

## 4. Causation

### 4.1. The Biopsychosocial Model

Young presented an integrated model of causality of behavior and psychopathology that focuses on the biopsychosocial model [101]. The biopsychosocial model is a heuristic one that refers to a compendium of multiple factors that influence and induce psychological disorders and conditions. The model is still current and has been used to help explicate depression [102]. PTSD is also a disorder that fits the biopsychosocial model [103]. The literature on chronic pain refers to the multifactorial biopsychosocial causal model [101].

The advantage of using the biopsychosocial approach therapeutically was underscored by Young [104]. Young organized all the empirically supported change mechanisms in youth psychopathology after events such as trauma into an integrative model by characterizing the techniques found effective as one or the other three components of the biopsychosocial term. Clearly, the model can inform therapy for biopsychosocial-related conditions such as PTSD and chronic pain [104].

### 4.2. Scale

D’Angelo and Jirsa described cross-CNS structure and function from a multiscale perspective in a model that includes several major scales, from the micro to the macro and including middle-level ones referred to as mesoscales [105]. The bottom–up microscale levels refer to neurons and local microcircuits, referred to as elemental causes in this inclusive model. The upper-level macroscale levels refer to the connectome and ensembles or assemblies in brain organization.

According to D’Angelo and Jirsa, the different organization scales in brain organization can co-exist in activity in a mixed, intersecting fashion, demonstrating integration. Downward directions in the system are direct, and upward ones are inverse [105]. The brain is organized to buffer injury and disease, having redundance, resilience, and plasticity mechanisms, such as neuro-degeneracy. D’Angelo and Jirsa refer to models in system organization that apply to their multiscale brain model, such as nonlinear dynamic systems theory (applied to synaptic organization) and network theory (with nodes/core units and edges/links), applied to neural mass and connectome modeling [105]. Aside from brain multi-scaling, the authors refer to time scales, for example, in learning and development. They refer to coupling in system organization, both global and specific. They refer to subsystems in the connectome or “subject-specific connectomes”. They refer to the balance in neuronal activity in excitation and inhibition. As for task-driven behavior, they refer to functional activity and control systems in sensorimotor (e.g., embodied) and cognitive modeling. They do refer to parameterization (e.g., collective variables) and virtual brain models, but they admit aspects, such as development and pathology, are hard to integrate into the overall model.

Suárez et al. describe the connectome in more detail, and they articulate how functional connectivity in the brain emerges from task-dependent brain activity and underlying structural connectivity [106]. They consider different scales in brain organization, from the neuron to broad cytoarchitecture. The brain is hierarchically organized, with nested, embedded heterogenous structure–functional connections or couplings. This arrangement supports perception, cognition, and action. Neuronal co-activation can take place inter-regionally or among more distant, spatially distributed neuronal activity centers. Neuronal ensembles involve coordinated neural activity. They form structural and functional communities having perceptual, cognitive, and affective relevance. Cortico–cortical and ascending arousal-based projections engage in segregating or integrating modulatory signal exchange. Network theory helps specify the nature of links in the connectome, for example, referring to the density of connections as expressing high local clustering and short path length, which is referred to as “small world” architecture. Nodes form “hubs”, and hubs form “rich clubs”. Young hypothesized that the left hemisphere is more tightly organized in terms of these network science concepts, consistent with its intricate specializations manually and in language, for example [107].

The research on connectomes in brain modeling refers to implications for perception, cognition, affect, and behavior (action) generally. The reviewed articles refer to control systems and concepts involving activation (e.g., signally integration, neuronal excitation) and inhibition (implicit in segregation).

### 4.3. Reception to Conception

Damasceno describes the relation among reception, sensation, perception, and conception. Receptors are peripheral primary sensory neurons [108]. Receptor regions in the skin or retina consist of not only excitatory neurons leading to the spinal cord but also inhibitory neurons acting in conjunction with them to create second-order excitatory neurons in the thalamic transmission to the brain. Sensation deals with adducing isolated properties of received signals related to external objects or phenomena after the stimulus excitation of receptor cells. Sensation takes place in modality-specific sensory–perceptual (perceptive) analyzers in the cerebral cortex. Sensory–perceptual analyzers involve the fine-tuning of the receptor organs as well as synthetic activity across the spectrum of components in the reception to cognition reciprocal interaction. In perception, the process ascribes meaning to the sensed object/phenomenon, which requires complex mental operations. Perception is a co-constructed relational activity with language involved, as in naming objects.

Thórisson et al. considered perception and cognition so intertwined that they referred to their integration in the concept of cognitive perception [109]. Cognitive perception refers to the coordination between perception and “knowledge-based control, learning and general cognition” [109] (p. 1).

Tsur and Talmon have an integrated concept for trauma on “orientation to bodily signals”. [110]. For these authors, trauma is powerful and insidious, leading to catastrophizing, hypervigilance, and fear about the body and its function. This process will lead to negative, frightful perceptions and interpretations of bodily signals. The body becomes the “stage” or somatic channel of traumatic event memory storage on which negative experiences can act, and the process is accentuated by the psychophysiology in trauma reactions/PTSD. The process of orientation to bodily signals fits well with the present approach on the reciprocal interactions among reception, sensation, perception, and conception, or in this case, cognitive interpretation. Oliviera et al. referred to a similar concept of interoceptive sensibility in relation to pain [111].

### 4.4. Psychological Control

The concepts of psychological reserve and psychological control were developed by Young in relation to trauma (e.g., child maltreatment/abuse) and PTSD [107,112]. Psychological reserve refers to the factors that affect the ability to resist stress and reduce feeling overwhelmed, such as maintaining a healthy lifestyle and knowing how to cope with stress. The capacity to resist (cope with) the effect of a stressor is buttressed by having a good psychological reserve, which can be depleted by poor sleep, health factors, lifestyle factors, etc.

Psychological control refers to having a sense that one is in control of the context and not being overwhelmed. Young proposed that psychological disorders and impairments are affected by a sense of loss of psychological control of the environment. The loss of a sense of having psychological control is worsened by psychological reserve depletion. The interactive psychological reserve/psychological control process can be accentuated by the stress response including the fight/flight/hide response, which is referred to as a loss of internal control. Therefore, in Young’s model, when there is a loss of internal control and a perception of a loss of external control, a cascade effect might be potentiated. When this vicious circle happens, it can exacerbate symptoms and impairments. As well, the person might be psychologically predisposed to psychologically exaggerate symptoms and dysfunctions. For example, in cases of PTSD, the person feels overwhelmed and out of control of their environment, and the psychological reserve that could help control this feeling is depleted by the cascading events of the experienced trauma, for example, poor sleep. Furthermore, it could be argued that the pain experience and associated fatigue deplete the psychological reserve and potentiate lack of a sense of psychological control associated with the continual pain experience.

Some research has dealt with analogous concepts to that of having a sense of psychological control. Peck et al. studied the relationship between cued cognitive control to trauma recollection and PTSD [113]. The cued cognitive control task is only partially related to psychological control as presently defined. The former was measured using a common executive function task on card sorting (Wisconsin Card Sorting Test (WSCT)). The study participants were followed for three months after their MVA. Poorer cognitive control (poorer resistance to interference on the WCST) emerged as a risk factor for PTSD in the study.

As for pain, Palyo and Beck examined perceived life control in relation to PTSD in participants injured and with pain as well after their MVAs (which had taken place on average 28 months previously) [114]. Perceived life control was measured by the perceived life control subscale of the Multidimensional Pain Inventory (MPI) [115]. The subscale consists of four items, with three of them addressing a sense of loss of psychological control as defined presently (related to problem solving, coping with stress, and having control over your life; the fourth item concerns control over pain). The authors found that perceived life control was related to PTSD symptoms, pain severity, and psychosocial impairment. Hass et al. tested active-duty military participants with PTSD after cognitive processing therapy (N = 127) [116]. The participants who responded to therapy demonstrated better pain-related and health-related outcomes, including for perceived life control, compared with therapy non-responders.

### 4.5. Activation–Inhibition Coordination

Young described how activation and inhibition work in concert in the reaching and grasping of one-month olds [107]. He showed that the right hand of these infants performed better in a midline reaching task compared with the left hand. He related the infant right-hand skill to the developing left hemisphere specialization for coordinated motor behavior; furthermore, he inferred that the underlying mechanism related to a better activation–inhibition coordination in the left compared with the right hemisphere innately from birth. The example of the left hemisphere becoming specialized for speech fits this conception, in that speech is a complex motor task. Young reviewed the literature to indicate that activation–inhibition coordination processes are widespread and are cross-scale over neuron, brain, and behavior despite that terminology not being used directly in the research [107]. For example, for neurons, the literature refers to the optimal ratio of excitement to inhibition (also see Babik and Lobo and He et al., who reference the activation–inhibition coordination concept) [117,118].

Table 4 summarizes the major findings of the review on the general causes of psychological injuries. It shows that the causal factors in the expression of psychological injuries cover the full spectrum of biopsychosocial factors. Those include the posited psychological reserve and psychological control. A general mechanism across the brain–behavior axis might involve activation–inhibition coordination.

Next, this article presents an integrated model of generalized sensitization in the major psychological injuries. The model focuses on (a) a broad range of loci of sensitizations, (b) a broad range of conditions (e.g., not just chronic pain but also PTSD and MTBI/concussion), and (c) possible applications in therapeutic practice, focusing on sensitization and related bodily registers of trauma. The model builds on the first part of this article on the neurobiology of psychological injuries.

## 5. Sensitization Model of Psychological Injury

**Figure 1:**
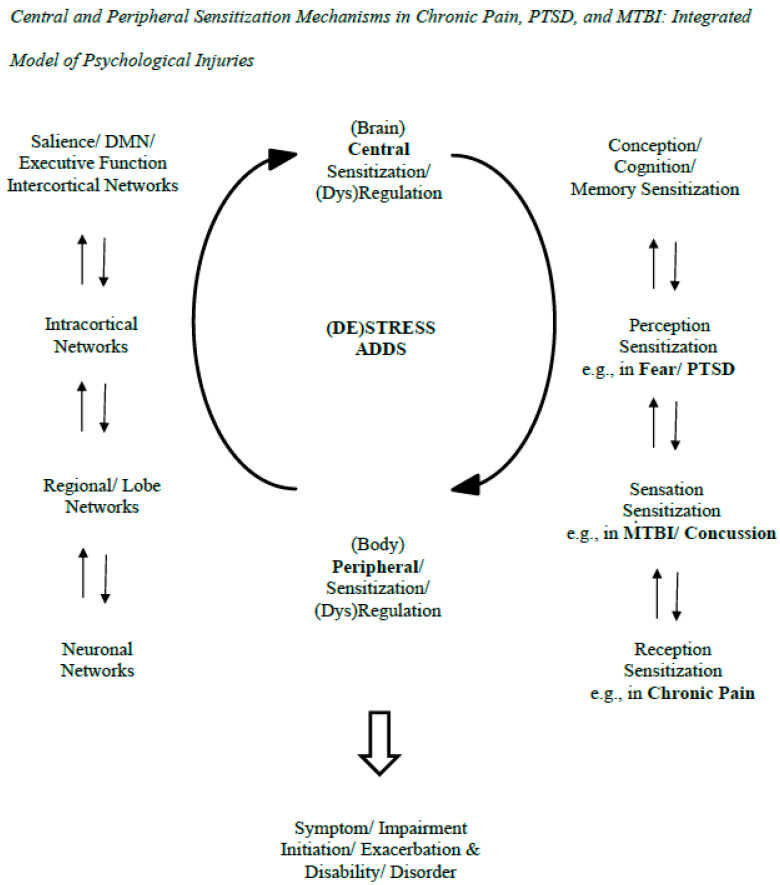
This figure illustrates how brain/central and bodily/peripheral functions and their sensitivities/dysregulations mutually interact in the generation of symptoms and exaggerations/exacerbations of psychological symptoms that take place after negligent events at claim. These processes can apply equally to PTSD and MTBI symptoms and not only chronic pain ones. The central part of the figure depicts the interaction of central and peripheral sensitizations in the process. The right part of the figure indicates the reciprocal interactions over different sensitizations related to reception, sensation, perception, and conception, which, respectively, relate to chronic pain, MTBI/concussion, PTSD/fear, and generalized sensitivity. The left side of the figure depicts the different levels of the CNS that are involved in the psychological injuries and their own reciprocal, dynamic interactions. The literature review on the neurobiology of the psychological injuries reviewed in the first part of this article supports these dynamic models of sensitization and the CNS generally depicted in the model. That said, the arrows in the model are not meant to represent formally established pathways that have been empirically supported in the present context; they indicate pathways requiring further precision, empirical support, and connection to the conditions/diagnoses at issue. Aside from further conceptualization and testing, the model in the figure needs research on its potential practical applications, including how sensitization-informed practice can help ameliorate patient conditions, providing adjunct added value to more standard and empirically supported approaches.

The model in the figure indicates the sensitization processes at play in psychological injuries (see Figure 1). The figure includes two interacting sensitizations (a. central; b. peripheral; Volcheck et al., 2023) [10]. Respectively, they refer to (a) neurons/neuronal networks that are more easily activated and expand in the brain and (b) bodily processes with similar properties (e.g., nociceptors in pain experience). Here, they are generalized to help explain PTSD and MTBI, or at least the latter’s persistence in PPCS. The sensitizations are expanded in the side panels to the left and to the right. On the left, the panel indicates the range of central reciprocal interactions from neuronal network to neuron. On the right, the panel indicates the reciprocal influences from peripheral to central sensitizations—they go from reception to sensation to perception to conception/cognition. Here, it is posited that fear/PTSD is associated more with perceptual sensitization while the effects of MTBI/concussion are associated more with sensory sensitization. Chronic pain is traditionally associated with receptor field sensitization. To generalize from pain-related nociception and peripheral sensitization, this would refer to somatization in general in various PPCS symptoms (e.g., headaches, dizziness). Also, it would refer to the perceptual priming/hypervigilant response in PTSD.

## 6. Discussion

The present model of central/peripheral sensitization as a common mechanism in psychological injuries adds to prior models by indicating the following. The model focuses on (a) a broad range of loci of sensitizations (e.g., reception, sensation, perception, conception), (b) a broad range of conditions (e.g., not just chronic pain but also PTSD and MTBI/concussion, and here we can add their comorbidities in polytrauma and related disorders, e.g., substance abuse disorder and depression), (c) hierarchical levels of explanation/causation (e.g., (i) a top–down level in the biopsychosocial model of compendium of factors, which includes patient perception and (ii) a bottom–up level of activation/inhibition coordination), (d) a detailed description of the neurobiology involved in the major psychological injuries that was undertaken as part of the literature review above, and (e) potential new avenues in instigating therapeutic change mechanisms (e.g., any relevant component of the broad biopsychosocial array, the specific activation–inhibition coordination, and increasing psychological reserve/control).

Therefore, central/peripheral sensitization appears to be a common mechanism in psychological injuries. When increasing central and peripheral sensitization as presently defined becomes a vicious circle, it could cause exacerbated symptoms and impairments. The forensic assessor needs to be aware of the full gamut of mechanisms involved in psychological injury, from common ones that exacerbate and exaggerate symptoms and impairments to legal-related ones in which motivations related to secondary gain are prominent. Understanding the role of sensitization in this process and the neurobiology involved brings insight into not only the difficulty in understanding the origins of the psychological injuries but also possible pathways in their treatment.

To summarize, the first part of this article examined the concepts of central and peripheral sensitization that is critical to understanding chronic pain, and it applied the concept to concussions/MTBI and fear/PTSD. This led to an expansion of the sensitization concept to sensory sensitization for concussion/MTBI and perceptual sensitization for fear/PTSD. Understanding the neurobiology of the major psychological injuries is required for the proper psychological assessment, diagnosis, and treatment of individuals expressing psychological injuries.

## 7. Conclusions

To conclude, psychological injuries involve neurobiological underpinnings, which is important to know for psychologists working in the field. That said, the literature review on the neurobiology of chronic pain, MTBI/concussion, and PTSD did not find definitive biomarkers of these psychological injuries. They are multifactorial, being influenced by a host of biopsychosocial factors. Assessors at court cannot use the argument that the conditions have biomarkers to justify their presence or enduring nature. Research should continue to demarcate general neurobiological underpinnings to chronic pain, MTBI/concussion, and fear/PTSD generally and in terms of sensitization specifically. Research should attempt to pinpoint pathways in the posited reciprocal sensitizations at the receptor, sensory, perceptual, and conceptual/cognitive levels.

The review of neurobiological factors for the major psychological injuries revealed common effects in sensitization, which has led to a sensitization model on relevant causal factors associated with psychological injuries. The sensitization model is embedded in a broader causal model of behavior that can be used in understanding them and changing them in psychotherapy. The models in this article are heuristic, like the biopsychosocial model in which they are embedded, and require targeted research. The field of psychological injury and law has few theoretical bases, aside from the biopsychosocial model, such that the present modeling constitutes an advance in the field [102].

## Figures and Tables

**Table 1:** Neurobiology of Chronic Pain.

Category	Literature Review Findings
Sensitization	
Central/peripheral	Both central and peripheral sensitization are involved; peripheral sensitization takes place especially at the receptor level.
Other Neurobiology	
Regions/Circuitry	Dorsal horn, amygdala, insula, nucleus accumbens, anterior cingulate cortex, secondary somatosensory cortex, prefrontal cortex, fronto–parietal cortex, cingulo–parietal–frontal cortex.

**Table 2:** Neurobiology of Mild Traumatic Brain Injury/Concussion.

Category	Literature Review Findings
Sensitization	
Central/peripheral	Both central and peripheral sensitization appear involved; sensitization takes place especially at the sensory level.
Other Neurobiology	
Regions/Circuitry	Perceptual regions, salience, attention networks, default mode network, executive control network, visual, auditory networks.

**Table 3:** Neurobiology of Fear/Posttraumatic Stress Disorder.

Category	Literature Review Findings
Sensitization	
Central/peripheral	Both central and peripheral sensitization appear involved; sensitization takes place especially at the perceptual level.
Other Neurobiology	
Regions/Connectome	Thalamus, cerebellum, limbic system, amygdala, hippocampus, insula, locus coeruleus, anterior cingulate cortex, prefrontal cortex, central executive network.

**Table 4:** Generalized Causality of Psychological Injuries.

Category	Literature Review Findings
Biopsychosocial Model	
Multifactorial	Psychological injuries are multifactorial, with no biomarkers, and their causal components interact at multiple levels.
Psychological Reserve/Psychological Control	
Reserve/Control	Psychological injuries are expressed when reserve is low and a sense of having no control in the environment is high.
Activation/Inhibition Coordination	
Activation/Inhibition	This coordination takes place at multiple levels of the brain/behavior axis: from neuron to neuronal circuitry to behavior.

## Data Availability

Not applicable. Data sharing is not applicable to this article, as no datasets were generated or analyzed during the current study.

## Abbreviations

The following abbreviations are used in this manuscript:

Abbreviation	Term
ACT	Acceptance and Commitment Therapy
ACRM	American Congress of Rehabilitation Medicine
ACC	Anterior Cingulate Cortex
ANS	Autonomic Nervous System
CEN	Central Executive Network
CNS	Central Nervous System
CPTSD	Complex Post Traumatic Stress Disorder
CRPS	Complex Regional Pain Syndrome
DMN	Default Mode Network
DSM-5-TR	Diagnostic and Statistical Manual of Mental Disorders 5th ed Text Revision
DAI	Diffuse axonal injury
DSO	Disturbances in self-organization
EAET	Emotional Awareness and Expression Therapy
GABA	Gamma-aminobutyric Acid
GCS	Glasgow Coma Scale
HPA	Hypothalamic–pituitary–adrenal
IASP	International Association for the Study of Pain
ITQ	International Trauma Questionnaire
LOC	Loss of Consciousness
MTBI	Mild Traumatic Brain Injury
MBI	Mindfulness-Based Interventions
MVA	Motor Vehicle Accident
MPI	Multidimensional Pain Inventory
MESSY	Multi-Modal Evaluation of Sensory Sensitivity
NA	Nucleus accumbens
PNS	Peripheral Nervous System
PPCS	Persistent post-concussion syndrome
PTA	Post Traumatic Amnesia
PTSD	Post Traumatic Stress Disorder
PFC	Prefrontal Cortex
SSD	Somatic Symptom Disorder
SNS	Sympathetic Nervous System
TKA	Total knee arthroplasty
WCST	Wisconsin Card Sorting Test
